# Novel insights regarding the measurement properties of the SCOPA-AUT

**DOI:** 10.1186/s12883-022-03008-2

**Published:** 2022-12-13

**Authors:** Albert Westergren, Klas Wictorin, Oskar Hansson, Peter Hagell

**Affiliations:** 1 The PRO-CARE Group, and The Research Platform for Collaboration for Health, Faculty of Health Sciences, Kristianstad University, Kristianstad, Sweden; 2 Department of Neurology, Helsingborg Hospital, Helsingborg, Sweden; 3 Department of Clinical Sciences, Lund University, Lund, Sweden; 4 Department of Clinical Sciences, Lund University, Malmö, Sweden; 5 Memory Clinic, Skåne University Hospital, Malmö, Sweden

**Keywords:** Autonomic dysfunction, Measurement, Parkinson’s disease, Rasch model, Response categories

## Abstract

**Background:**

The Scale for Outcomes in Parkinson’s disease for Autonomic symptoms (SCOPA-AUT) is an instrument intended to assess overall and domain-specific autonomic symptom burden. In this study the SCOPA-AUT is translated into Swedish and its measurement properties are assessed.

**Methods:**

Following translation the SCOPA-AUT was field-tested regarding comprehensibility, relevance, and respondent burden (*n* = 20). It was then tested according to Rasch measurement theory using data from 242 persons with PD, of whom 162 completed SCOPA-AUT at baseline and 1–2 years later, giving a total of 404 data points for analysis.

**Results:**

The Swedish SCOPA-AUT took a mean of 6 min to complete and was considered easy to use and relevant by respondents. SCOPA-AUT exhibited acceptable Rasch model fit, represents more severe levels of dysautonomia than that reported by the sample, and response categories were not working as expected for 17 items. Local dependency was identified and followed a pattern resembling the suggested subscales. Accounting for the subscale structure eliminated local dependency and reduced the initially inflated reliability from 0.81 to 0.68.

**Conclusions:**

The SCOPA-AUT is useful as a clinical check-list but requires further developmental work in order to meet more rigorous standards as an outcome measurement instrument.

**Supplementary Information:**

The online version contains supplementary material available at 10.1186/s12883-022-03008-2.

## Background

Parkinson’s disease (PD) is a progressive neurodegenerative disorder characterized by motor symptoms (bradykinesia, rigidity and tremor), but non-motor symptoms, e.g., cognitive dysfunction, anxiety, depression, sleep dysfunction, pain, and dysautonomia, are also common [1] and need to be considered in the clinical assessment of PD and therapeutic outcomes [2, 3]. Dysautonomia is not only a feature of PD, but is also common in e.g., multiple system atrophy and other atypical parkinsonian syndromes. One commonly used tool developed specifically to assess dysautonomia in PD and related disorders, is the SCales for Outcomes in PArkinson’s disease for Autonomic symptoms (SCOPA-AUT) [4].

The SCOPA-AUT was developed based on a literature review of autonomic symptoms in PD and multiple system atrophy and input by specialised clinicians [4]. The scale yields a total dysautonomia score, as well as six subscale scores. Studies using classical test theory have generally found SCOPA-AUT scores to be reliable and valid for assessing autonomic dysfunction in PD [4–7]. While traditional methodologies such as classical test theory are commonly used, Rasch measurement theory (RMT) [8, 9] is considered superior in terms of testing the extent to which rating scales are appropriate as outcome measures [10]. In the case of the SCOPA-AUT, one previous Spanish study used RMT to assess its measurement properties [11] and found some weaknesses, such as compromised scale-to-sample targeting (the sample reported less severe dysautonomia than that represented by the scale), differential item functioning by age and gender, signs of item redundancy, and evidence that response categories did not work as expected. In addition, the authors questioned the use of subscale scores since they found indications that the full SCOPA-AUT represents a single variable [11]. However, the authors did not explicitly take the subscale structure of the SCOPA-AUT into account in their analyses. Furthermore, since no other study appears to have used RMT to investigate the SCOPA-AUT, it is unknown to what extent the observations by Forjaz et al. [11] are generalizable. Therefore, we reassessed the SCOPA-AUT as a unidimensional measure of dysautonomia in people with PD using RMT with data from the Swedish version of the scale. In addition, we also describe the translation of the SCOPA-AUT into Swedish.

## Methods

The studies were conducted in accordance with the Declaration of Helsinki (1964) and its later amendments. The studies were approved by the Regional Ethics Committee in Lund, Sweden (Kristianstad University: 2009/429 and 2009/226; Lund University: 2008/290). All participants provided written informed consent prior to their inclusion. The data that support the findings of this study are available from the corresponding author upon reasonable request.

### Translation and field-testing of the Swedish SCOPA-AUT

SCOPA-AUT was translated into Swedish by means of the dual-panel method [12]. It was first translated from English into Swedish by a bilingual panel of five individuals fluent in both languages, who produced a consensus translation. A second panel (six lay people) reviewed the translated version to ensure it was expressed in natural, everyday language. The Swedish SCOPA-AUT was then field-tested regarding comprehensibility, relevance and respondent burden (time taken to complete the questionnaire) using a convenience sample of 20 persons with PD (15 men; mean age, 67.5 (Standard Deviation (SD), 6.4) years; mean PD duration, 9 (SD, 5.1) years).

### Psychometric testing of the Swedish SCOPA-AUT

#### Sample

In the psychometric testing of the SCOPA-AUT, persons with PD [13] without clinically significant cognitive impairment (as determined by their attending clinician and routine cognitive screening) were recruited consecutively when they had appointment at any of two PD clinics. In addition, persons with conditions that made it difficult to participate (e.g., terminal cancer) were excluded. Of 404 available assessments, 242 were conducted at baseline and 162 at follow-up, 1–2 years later; 268 of the assessments were conducted at a university hospital (from the prospective Swedish BioFINDER study, http://www.biofinder.se), and 136 at a regional hospital as part of a longitudinal observational study [14]. At baseline (*n* = 242), the mean (SD) age was 66.2 (9.9) and 147 (60.7%) were men. The median time since diagnosis was 4 (q1-q3, 1–8) years and all five Hoehn & Yahr stages [15] were represented (median, 2; q1-q3, 1–2.5).

Clinical data were collected by means of clinical examination by a movement disorder specialized neurologist or PD specialized nurse, and SCOPA-AUT data were collected by means of self-report, during the “on” phase (i.e., periods with good antiparkinsonian drug response).

#### SCOPA-AUT

The SCOPA-AUT [4] assesses autonomic functioning by instructing respondents to indicate how often they experience the problems defined by each of its 25 items according to four ordered response categories (never [0], sometimes [1], regularly [2], and often (3]). Item scores can be summed into six subscale scores representing gastrointestinal (7 items), urinary (6 items), cardiovascular (3 items), thermoregulatory (4 items), pupillomotor (1 item), and sexual (2 items for men and 2 items for women) functioning. In addition to the four ordered response categories, urinary and sexual functioning items have the additional response categories “use catheter” and “not applicable”, respectively. Given the gender specific sexual functioning items, each respondent answers 23 items. In addition to the six subscales, a total dysautonomia score (sum of all item scores; possible range, 0–69) has been proposed. In all instances, higher scores represent more autonomic dysfunction.

#### Analyses

The analyses followed the procedure by Forjaz et al. [11] regarding the additional response categories for urinary and sexual functioning items, as well as for the gender specific sexual functioning items. That is, response categories “use catheter” (urinary functioning) and “not applicable” (sexual functioning) were treated as missing values. Furthermore, the four female and male sexual functioning items were pooled so that male erection problem (item 22) and female vaginal problem (item 24) were treated as one item (re-named “sexual arousal problems”). Similarly, male ejaculation problems (item 23) and female problems with orgasm (item 25) were treated as one item (re-named “orgasmic problems”).

SCOPA-AUT data were analysed according to the unrestricted (“partial credit”) polytomous Rasch model using RUMM2030 (Professional Edition 5.4) [16]. *P*-values are two-tailed and considered significant when < 0.05 following Bonferroni adjustment. The analyses addressed targeting, reliability (the Person Separation Index, PSI, and coefficient alpha), response category functioning, Rasch model fit, uniform and non-uniform Differential Item Functioning (DIF) by time of assessment (baseline vs. follow-up), age (subgroups according to median age) and gender, and local dependency (where the SCOPA-AUT subscale structure was taken into account by the creation of subtests) [9, 17]. DIF by time of assessment was checked at the outset of these analyses and absence of DIF by time was taken as support for merging data from the two time points, thereby gaining precision in estimates [18]. A detailed description of the analysis is given in the additional text file (see Additional file 1), and main concepts related to the analysis are explained in Table 1.

**Table 1:** Explanations of concepts used in the analysis

Concept	Explanation
Coefficient alpha	An index of reliability (internal consistency)
Differential Item Functioning (DIF)	Assessment of weather items work invariantly in different subgroups of respondents, e.g., age and gender groups
Fit residual	Quantification of differences between observed and expected item responses. The expected value is 0 (perfect fit) and values between -2.5 and + 2.5 are considered acceptable
Item Characteristic Curve	Displays the relationship between observed and expected item responses at various levels, from less to more dysautonomia. This is one aspect of Rasch model fit (see below)
Local dependency	Occurs when there is item redundancy (different items represent similar content), or can be due multidimensionality (see below)
Multidimensionality	Items do not represent one common construct
Person separation index (PSI)	An index of reliability that is conceptually analogous to coefficient alpha (see above)
Rasch model fit	Different related approaches to assess to what extend empirical data fit the Rasch model
Response category functioning	Weather response categories (for example never [0], sometimes [1], regularly [2], and often [3]) function as intended. Response category thresholds (see below) that are disordered imply that response categories are not functioning as expected from less to more
Response category thresholds	The locations where there is equal probability of responding in either of two adjacent categories
Strata	The number of statistically distinct groups of persons that can be identified. For instance, with two strata, the scale can differentiate between persons with less versus more dysautonomia
Targeting	To what extent items in a scale represents the levels of the construct (in this study dysautonomia) reported by the sample
Unidimensionality	Items represents one common construct (in this study dysautonomia)

## Results

### Field-testing of SCOPA-AUT

Respondents (*n* = 20) completed the SCOPA-AUT in a mean of 6.0 (SD, 2.2; min–max, 2–10) minutes. The SCOPA-AUT was considered easy to understand (*n* = 20), answer (*n* = 17) and to be relevant (*n* = 18). Since no respondent comments concerned the translation, no modifications were made.

### Psychometric testing of SCOPA-AUT

There was no DIF by time for data collection (baseline and follow-up). Data from the two time points were therefore merged in the analyses.

**Fig. 1:**
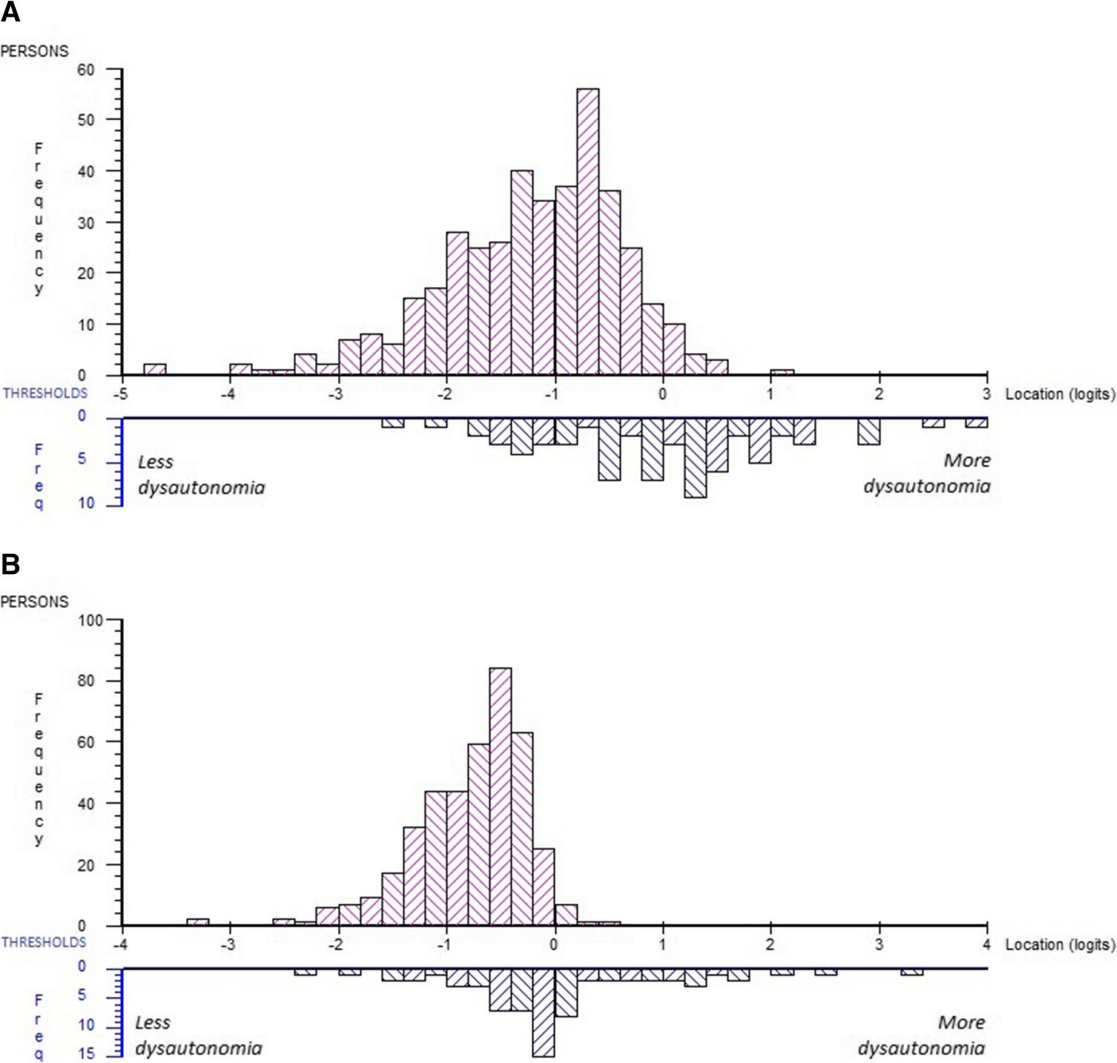
Person-item threshold distribution, distribution of people (upper panels) and response category thresholds (lower panels) on the common logit metric (x-axis; positive values = more autonomic dysfunction), in Panel **A** for the 23 SCOPA-AUT items, and in panel **B** for the SCOPA-AUT six subscales

The mean person location was -1.215 (SD, 0.845) indicating that the SCOPA-AUT tends to represent more severe levels of dysautonomia than that experienced by the sample (Fig. 1A). There was no ceiling effect, i.e., no person reported the highest (worst) possible score on SCOPA-AUT, and a 0.5% floor effect (2 persons scored 0). There was a significant difference (*P* < 0.001) in mean person locations between those with mild (Hoehn & Yahr stage ≤ II; *n* = 296) and those with moderate/severe functional disability (Hoehn & Yahr stage > II; *n* = 108), mean (SD) person locations -1.334 (0.842) and -0.891 (0.769) respectively. The PSI for the total SCOPA-AUT was 0.81 (alpha, 0.84), implying that three (i.e., 3.12 strata) distinct levels of dysautonomia can be identified [19].

Response category thresholds worked as intended for six items, whereas disordered thresholds were evident in the remaining 17 items (items 1–4, 6–10, 12, 13, 16, 18, and 19). The general pattern was that either one or both middle response categories (“sometimes” and “regularly”) were less prone to appear as the most probable responses.

**Fig. 2:**
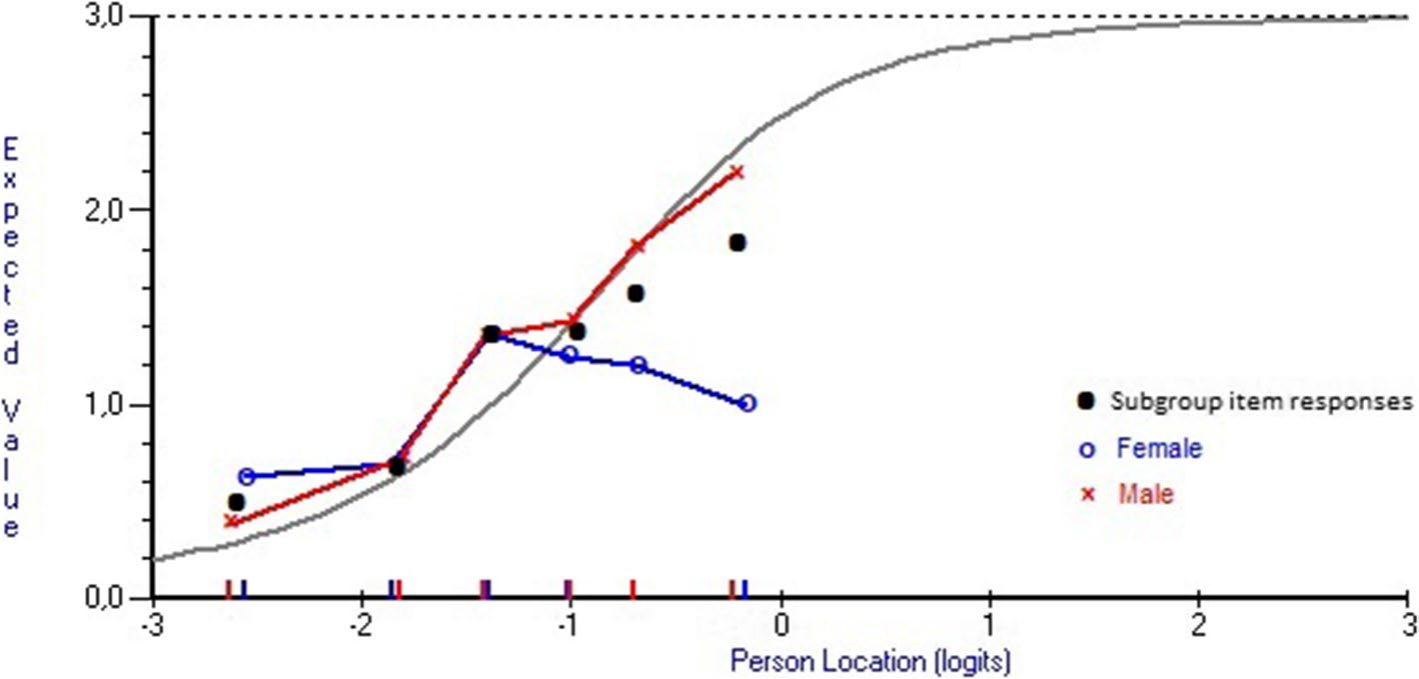
The item characteristic curve (ICC; grey curve) representing expected item responses (y-axis) at various levels of dysautonomia (x-axis) for item 22 (“sexual arousal problems”). Black dots are empirical item responses by subgroups of persons with various levels of dysautonomia (markers on the x-axis) and illustrates an underdiscriminating response pattern. When divided by gender (men = red curves/markers, women = blue curves/markers) it is seen that despite increasing levels of dysautonomia (SCOPA-AUT total scores), women tend to have unchanged or decreasing problems with “sexual arousal” while the plot for men follow the expected ICC

Table 2 lists item locations (each item location is the mean of its response category threshold locations) where negative values represent less autonomic dysfunction and positive values represent more autonomic dysfunction. There were no major deviations from model expectations, indicating general item level model fit (Table 2). Only two items had a fit residual exceeding ± 2.5 and that was “Sexual arousal” (item 22; fit residual, 3.519) and “Urinary incontinence” (item 9; fit residual, -2.519). The item characteristic curve (ICC) for item 22 shows that this item tends to underdiscriminate, i.e., it may not represent the same construct as the test as a whole (Fig. 2). Taking gender into account suggests that despite increasing levels of dysautonomia, women tended to have unchanged or even less problems with “sexual arousal” (item 22) while the plot for men followed a more expected pattern.

**Table 2:** SCOPA-AUT item level Rasch model location and fit statistics (complete cases only, *n* = 404) ^a^

*Domains* and items ^b^	Location (SE) ^c^	Fit Residual ^d^	Chi-square	*P*-value
*Gastrointestinal*				
1. Swallowing/choking	0.554 (0.085)	-0.500	1.153	0.949
2. Sialorrhea	-0.377 (0.065)	0.067	6.156	0.291
3. Dysphagia	0.702 (0.093)	-0.990	5.118	0.402
4. Early abdominal fullness	0.056 (0.072)	0.113	4.329	0.503
5. Constipation	0.152 (0.072)	-1.202	4.928	0.425
6. Straining for defecation	-0.476 (0.065)	-1.320	14.363	0.013 ^e^
7. Faecal incontinence	0.841 (0.122)	0.587	4.08	0.538
*Urinary*				
8. Urinary urgency	-0.378 (0.066)	-2.179	7.501	0.186
9. Urinary incontinence	-0.241 (0.067)	-2.519	10.466	0.063
10. Incomplete emptying	0.104 (0.075)	-0.958	4.95	0.422
11. Weak stream of urine	-0.219 (0.068)	-0.342	1.955	0.855
12. Frequency	-0.557 (0.069)	-0.710	5.325	0.377
13. Nocturia	-1.533 (0.058)	1.777	9.407	0.094
*Cardiovascular*				
14. Lightheaded (standing up)	0.245 (0.079)	-0.563	2.177	0.824
15. Lightheaded (standing some time)	0.898 (0.091)	-0.650	3.238	0.663
16. Syncope	1.432 (0.201)	-0.991	5.756	0.331
*Thermoregulatory*				
17. Hyperhidrosis during the day	0.159 (0.070)	-0.683	7.966	0.158
18. Hyperhidrosis during the night	0.150 (0.072)	0.094	2.691	0.747
20. Cold intolerance	-0.021(0.067)	0.825	8.94	0.111
21. Heat intolerance	0.202 (0.074)	0.389	7.386	0.193
*Pupillomotor*				
19. Oversensitive to bright light	-0.051 (0.070)	0.194	13.875	0.016 ^e^
*Sexual*				
22. Sexual arousal problems ^f^	-1.020 (0.068)	3.519	15.892	0.007 ^e^
23. Orgasmic problems ^g^	-0.622 (0.073)	-0.236	4.654	0.459

^a^ Excluding people (*n* = 2) with extreme scores (i.e., those scoring 0 and 69)

^b^ Items are presented by domain but were analyzed as a 23-item unidimensional scale

^c^ Item locations are expressed in logit values and represent the mean of each item’s response category threshold locations. Negative locations represent less autonomic dysfunction and positive locations represent more autonomic dysfunction

^d^ Standardized fit residuals represent discrepancies between observed and model-expected responses; should range between ± 2.5

^e^ Not significant following Bonferroni correction

^f^ Original items 22 (males; “erection problem”) and 24 (females; “vaginal problem”)

^g^ Original items 23 (males; “ejaculation problems”) and 25 (females; “problems with orgasm

There was no DIF by age, but significant uniform DIF by gender for items 2, 9, 11, and 21 (*P* < 0.001). Women were more likely to endorse items 9 and 21 (“urinary incontinence” and “heat intolerance”, respectively) while men were more likely to endorse items 2 and 11 (“sialhorrhea” and “weak stream of urine”, respectively). To resolve DIF, adjustment by item split for gender was needed in all four items, suggesting that all observed DIF were real and not artificial. However, gender DIF did not appear to bias person locations according to comparison between the DIF-adjusted scale and the original scale as judged by similar effect sizes for gender differences between the DIF-adjusted and the original scale (ESs, 0.146 (95% Confidence Interval (CI) -0.142, 0.435) and 0.214 (95% CI -0.075, 0.503), respectively), and an intraclass correlation coefficient of 0.99 between non-adjusted and DIF-adjusted scores.

There were 11 instances with significant local dependency as indicated by relative residual correlations (range, 0.19–0.61), as judged according to a critical value for relative residual correlations of 0.19 [20]. In general, the pattern of residual correlations coincided with the suggested SCOPA-AUT subscale structure. This strengthens the case for taking the subscale structure of the SCOPA-AUT into account by treating the suggested subscales as subtests in the analysis. We therefore combined items within the SCOPA-AUT subscales into six subtests and treated each subtest as a single item in the analysis. Among all persons (*n* = 404), including those with missing data the mean person location for the six subtests was -0.769 (SD, 0.517), again indicating that that the SCOPA-AUT tends to represent somewhat more severe levels of dysautonomia than that experienced by the sample (Fig. 1B).

Reliability (PSI) of the six subtests was 0.68 (compared to 0.81 with the 23-item SCOPA-AUT), implying that two (i.e., 2.29 strata) distinct levels of dysautonomia can be identified [19]. Fit statistics when analyzing the SCOPA-AUT as six subtests were acceptable (Table 3) and there was no DIF by either age or gender, and no noteworthy (range, 0.04–0.19; critical value, 0.23) relative residual correlations between the subscales, suggesting local independence.

**Table 3:** SCOPA-AUT subtest Rasch model location and fit statistics (complete cases, n = 225) ordered by location ^a^

Domains	Location (SE) ^b^	Fit residual ^c^	Chi-square	*P*-value
Sexual	-0.569 (0.039)	2.085	4.519	0.477
Urinary	-0.48 (0.019)	-1.278	4.776	0.444
Cardiovascular	0.213 (0.045)	0.364	6.686	0.245
Thermoregulatory	0.214 (0.029)	-0.624	12.095	0.034 ^d^
Pupillomotor	0.219 (0.067)	0.286	12.594	0.027 ^d^
Gastrointestinal	0.404 (0.022)	-2.139	12.536	0.028 ^d^

^a^ Excluding people (n = 2) with extreme scores (i.e., those scoring 0 and 69)

^b^ Item locations are expressed in logit values and represent the mean of each item’s response category threshold locations. Negative locations represent less autonomic dysfunction and positive locations represent more autonomic dysfunction

^c^ Standardized fit residuals represent discrepancies between observed and model-expected responses; should range between ± 2.5

^d^ Not significant following Bonferroni correction

Including only persons with complete SCOPA-AUT responses (*n* = 225), coefficient alpha was 0.66 when analysed as six subtests, compared to 0.84 when analysed as 23 individual items. In addition, the variance that is unique to the subscales (*c* = 1.046) and the latent correlation among the subscales (*r* = 0.477) were moderate. Most of the systematic variance was left as non-error common variance (*A* = 0.783). (See the Supplementary information file for details regarding these indices.) The subtest overall test-of-fit (*P* = 0.021) was somewhat worse compared to the discrete 23 items (*P* = 0.178). These results could be suggestive of some potential violation of unidimensionality. Unidimensionality was therefore further tested by comparing person locations based on subtests representing the least severe (sexual/urinary; see below) with person locations estimated from the subtest representing the most severe levels of dysautonomia (gastrointestinal) using the independent t‐test approach [21, 22]. This identified 6 persons (2.7%; 95% Agresti-Coull CI, 1.1–5.8%) with significantly different locations when estimated from the sexual/urinary subtests compared with the gastrointestinal subtest. This supports unidimensionality since the proportion of persons with different estimates is less than 5%.

**Fig. 3:**
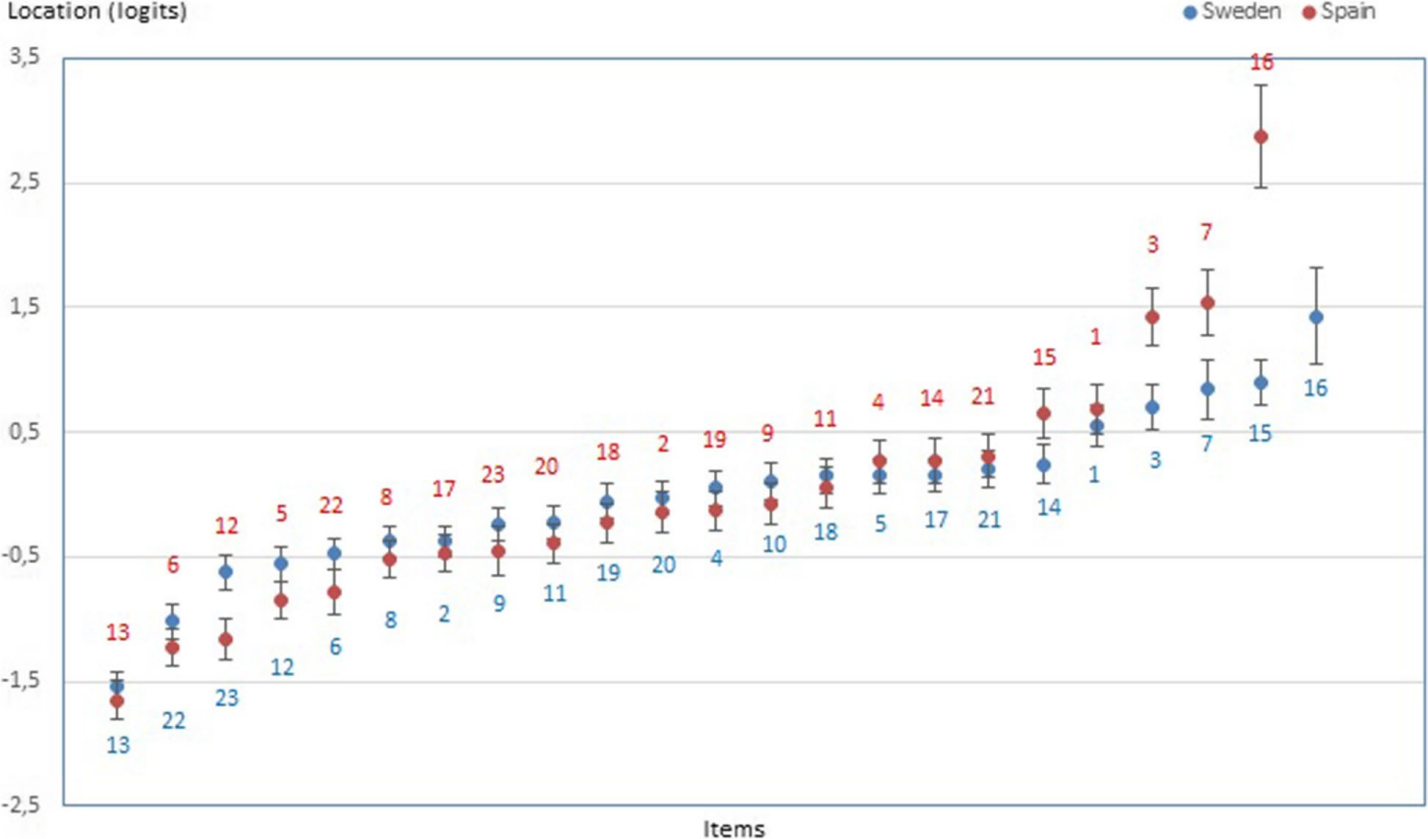
Hierarchical item ordering with item locations (± 95% CIs) on the y-axis (negative values = less dysautonomia) in this study (Sweden; blue dots/numbers) and a previous Spanish study [11] (Spain; red dots/numbers). Item 10 was excluded in the Spanish study [11]. Significant differences in item locations between the Spanish and this study were found for items 3, 5–7, 12, 16–18, and 20. Items are hierarchically ordered from the easiest (to the left) to the most severe (to the right) items.

The relative locations of the six SCOPA-AUT subtests (Table 3) suggest that sexual and urinary problems are the easiest to endorse and that gastrointestinal represent the most severe domain. Because Forjaz et al. [11] did not consider SCOPA-AUT domains, we cannot relate these findings to theirs. However, at the item level, locations are generally similar across the two studies (Pearson’s *r*, 0.882), with “nocturia” (item 13) at one end and “syncope” (item 16) at the other end of the severity continuum (Fig. 3).

## Discussion

The Swedish SCOPA-AUT was found to be relevant and easy to understand and use by people with PD, and respondent burden was acceptable. In addition, our psychomtric findings largely mirror, but also expand on those in the RMT based Spanish study [11], showing generally good fit to the Rasch model. However, limitations with the SCOPA-AUT were also identified, i.e., items represent more severe dysautonomia than that reported by the sample, response categories do not work as intended in a majority of items. Furthermore, there was minor DIF by gender in four items, as well as local dependency that could be resolved by taking account for the subscale structure in the analysis.

Similarly to the study by Forjaz et al. [11], the sample did not represent the full range of item locations with few persons reporting more severe dysautonomia. Our sample also appear to represent people with somewhat milder dysautonomia compared to those in the study by Forjaz et al. (mean location -1.434 and -1.068 respectively) [11]. This implies two things. First, both studies have some limitations in the ability to assess the measurement properties of the SCOPA-AUT, particularly towards the upper end of the scale. Future studies of the SCOPA-AUT should therefore attempt to include persons with more severe dysautonomia in order to better evaluate the full instrument. Second, persons with PD who report low levels of dysautonomia are not measured very well by the SCOPA-AUT. While it may be argued that this is of less concern and it is more important to be able to capture those with more pronounced problems (who arguably also would be the ones primarily targeted by various therapies) it remains a measurement limitation of the instrument.

We found reversed response category thresholds in a majority of SCOPA-AUT items, indicating that the response scale does not work as intended and that the current scoring may be unjustified [23]. The most likely reason for the disordered thresholds is that respondents fail to discriminate between two adjacent categories (“sometimes” or “regularly”). In accordance with our observations, Forjaz et al. [11] also found disordered thresholds for most items of the Spanish SCOPA-AUT. The significance of disordered thresholds is under debate [24–28]. However, since the ordering of categories reflects the respondents understanding of what it means to have more or less of the property and categories are assumed to be operating as intended, evidence of proper ordering is critical [27]. Since the same response scale is used across all items the threshold reversal is probably generalizable rather than incidental [23]. Thus, available data presented here and elsewhere [11] suggest that revision of the SCOPA-AUT response categories should be considered, either by reducing the number of response categories and/or by rewording category wording.

One item (22, “sexual arousal”) exhibited poor model fit. Inspection of item responses relative to its expected ICC revealed a divergent pattern among women while the responses for men followed what was expected. That is, despite increasing dysautonomia severity, women tended to have unchanged or even decreasing problems with “sexual arousal”. Other studies have reported similar findings, i.e. despite an increase in all SCOPA-AUT items with disease severity there is no increase in sexual dysfunction among women [4, 7], and another study found no association between sexual dysfunction and disease severity among women while it was significant among men [6]. Regardless of the explanation for these observations, they suggest that this item should not be merged but kept separate for men and women.

There was no DIF by age but four items exhibited evidence of DIF by gender. In two items women were more likely to endorse a positive response and in the other two it was the opposite. This probably contributed to the relatively minor importance of the observed DIF and the fact that there was no DIF when the SCOPA-AUT was analyzed as six subtests. It has been argued that if external information facilitate the understanding and interpretation of real DIF, resolving DIF may threaten content validity [29]. In the case of SCOPA-AUT the DIF found in four items can be clinically justified. That is, heat intolerance (item 21) and urinary incontinence (item 9) would be expected to be more common among women due to, e.g., menopause and weakened sphincter muscles, respectively, and weak stream of urine (item 11) would be expected to be more common among men due to prostate enhancement. This has implications for how best to deal with these instances of DIF [30]. If these problems are considered important for the measured variable (dysautonomia), regardless of whether they appear due to autonomic dysfunction or because of other reasons (e.g., menopause), adjustment for DIF would compromise measurement validity [30]. Regardless, in this particular case the observed DIF appear to be of minor practical importance, particularly given the lack of DIF when taking the subscale structure into account, but should be kept in mind in future studies of the SCOPA-AUT.

As in the original item level analysis, there was good fit between data and the Rasch model when taking the subscale structure into account by creating subtests representing the suggested SCOPA-AUT subscales. However, the dispersion of person locations shrunk and reliability dropped considerably, which both are signs of the local dependency identified in the first analysis [31]. That is, the initial reliability estimates are inflated. This aspect of the measurement properties was not addressed in the previous RMT based study of the SCOPA-AUT [11]. Instead, the use of subscale scores was questioned due to support of unidimensionality of the full instrument [11]. However, this argument may be challenged because unidimensionality is a relative rather than an absolute concept that, among other things, depends on the conceptualization of the measured variable [32]. This may be illustrated by the metaphor of a rope, where a variable (e.g., dysautonomia) is thought of as a thick rope (cf. the SCOPA-AUT) that is made up by finer ropes (cf. subscales) that in turn consist of even finer threads (cf. items) [25]. Although our findings also partly support unidimensionality of the SCOPA-AUT, the measurement properties of its individual subscales remain to be examined.

The hierarchical subtest order, as determined by their respective locations and taking the respective uncertainties (95% CIs of locations) into account, revealed that sexual and urinary problems were easier than cardiovascular, thermoregulatory and pupillomotor symptoms, which in turn were easier than gastrointestinal problems. This pattern appears to make general sense from a clinical perspective and broadly corresponds with findings from a retrospective clinicopathological cohort study from the UK, where urinary problems were the most common and sweating abnormalities and upper gastrointestinal dysfunction were the least common, with orthostatic hypotension appearing in the mid-range [33].

Our PD sample represents people in relatively early stages of PD, also compared to those reported in a previous SCOPA-AUT evaluation [11]. However, our observations, including the observed item hierarchy, are in general agreement with those reported previously [11]. Thus, while generalizations to the PD population at large should be made with some caution, our findings suggests that the SCOPA-AUT is conceptually stable across Spanish and Swedish cultures/languages and appears appropriate for use in early PD. This is important since dysautonomia occur and increase throughout the disease trajectory [4, 33, 34], and useful scales need to be applicable in all stages of PD. However, further assessments of the measurement properties of the SCOPA-AUT should aim to include people with more severe PD and, particularly, with more pronounced dysautonomia in order to better elucidate its properties at the more severe end. Our data did not allow for assessment of test–retest stability. However, we did not detect any DIF by time, indicating that items work the same way over time. This often overlooked aspect is considered at least as important as test–retest stability, and a prerequisite for meaningful test–retest evaluation [17]. In addition, autonomic dysfunction in PD could be due to other conditions, which could not be distinguished in this study. However, the SCOPA-AUT is intended to assess dysautonomia and alert clinicians of the potential need for further examinations [4]. Therefore, we do not consider this a major limitation. A further limitation is that there were no other demographic or clinical details in common than those presented in the paper (i.e., age, sex, time since diagnosis, and Hoehn & Yahr stages), since data were combined from two different studies. Finally, our samples did not undergo any objective autonomic testing, which precludes analyses of the relationship between patient-reported and objectively measured dysautonomia.

## Conclusion

The SCOPA-AUT was found to be user-friendly by respondents and its total score appears to be a clinically useful indicator of autonomic symptom burden. However, it is also clear that the instrument can be improved, e.g., regarding its response categories. Furthermore, local dependency affects person measurement and artificially inflates reliability. In its current format, the SCOPA-AUT primarily appears to represent an autonomic symptom severity checklist rather than a rigorous measurement instrument. That is, at this stage its appropriateness as an outcome measure appears dubious. However, it appears be highly useful in clinical practice as a systematic means of detecting dysautonomia, or as a dysautonomia survey tool.

## Supplementary Information

**Additional file 1.**

## Data Availability

Regional Ethical Review Board in Lund placed certain restrictions on access to the data; therefore, the dataset in this study is only available upon written request to the responsible researcher (Albert Westergren).

## Abbreviations

CI: Confidence Interval
DIF: Differential Item Functioning
ES: Effect size
ICC: Item characteristic curve
PD: Parkinson’s disease
PSI: Person Separation Index
RMT: Rasch measurement theory
SCOPA-AUT: The Scale for Outcomes in Parkinson’s disease for Autonomic symptoms
SD: Standard Deviation
SE: Standard Error
