# Supplementary material for: Novel insights regarding the measurement properties of the SCOPA-AUT
Source: BMC Neurol. 2022 Dec 13;22:478. doi: 10.1186/s12883-022-03008-2 (PMC9746092; doi:10.1186/s12883-022-03008-2)
Supplement: Supplementary file 1 — Additional file 1. [file 12883_2022_3008_MOESM1_ESM.pdf]

Additional file 1

Novel insights regarding the measurement properties of the SCOPA-AUT

Albert Westergren [1] , Klas Wictorin [2, 3], Oskar Hansson [4, 5], Peter Hagell [1]

[1] The PRO-CARE group, and The Research Platform for Collaboration for Health, Faculty of Health Sciences, Kristianstad University, Kristianstad, Sweden.
[2] Department of Neurology, Helsingborg Hospital, Helsingborg, Sweden.
[3] Department of Clinical Sciences, Lund University, Lund, Sweden.
[4] Department of Clinical Sciences, Lund University, Malmö, Sweden.
[5] Memory Clinic, Skåne University Hospital, Malmö, Sweden

Corresponding author:
Albert Westergren
Faculty of Health Sciences
Kristianstad University
SE-291 88 Kristianstad
Sweden
E-mail: Albert.Westergren@hkr.se
Phone: +46 44 208550

Analyses of the SCOPA-AUT according to Rasch measurement theory (RMT)

SCOPA-AUT data were analyzed according to the unrestricted ("partial credit") polytomous Rasch model [1, 2] using RUMM2030 (Professional Edition 5.4). P-values are two-tailed and considered significant when <0.05 following Bonferroni adjustment.

According to RMT [3, 4] , the probability of a certain item response is a function of the difference between the level of the measured construct (e.g., autonomic dysfunction) represented by the item and that possessed by the person. The model separately locates persons and items on a common interval level logit (log-odd units) metric, ranging from minus to plus infinity (with mean item location set at zero). If data accord sufficiently with the model, linear measurement and invariant comparisons are possible [5, 6]. In the current analyses we addressed targeting, reliability, response category functioning, Rasch model fit, uniform and non-uniform Differential Item Functioning (DIF) by time of assessment (baseline vs. follow-up), age (subgroups according to median age) and gender, and local dependency. DIF by time of assessment was checked at the outset of these analyses and absence of DIF by time was taken as support for merging data from the two time points, thereby gaining precision in estimates [7]. Analyses include both graphical as well as statistical methods, which are of equal primacy.

Good ***targeting*** means that items represent the levels of autonomic dysfunction reported by the sample and, conversely, that the sample distribution covers the levels of autonomic dysfunction represented by the items. Poor targeting compromises measurement precision, and conditions for scale evaluation [6]. One indicator of targeting is the average person locations relative to item locations (i.e., 0 logits) [6].

Reliability was estimated through the Person Separation Index (PSI), which is conceptually analogous to coefficient alpha [8] and can be used to derive the number of strata (i.e., statistically distinct groups of persons) that can be distinguished by the scale [9-11]. In addition, we also report coefficient alpha.

Whether the four ordered SCOPA-AUT response categories (never [0], sometimes [1], regularly [2] and often (3]) function as intended was assessed by studying response category ***thresholds***, i.e., the locations where there is equal probability of responding in either of two adjacent categories.

Disordered thresholds imply that response categories are not functioning as expected from less to more [12].

Model fit was assessed by several related approaches that concern standardized item fit residuals, which represent the discrepancies between observed and model-expected item responses [1]. Graphically, item characteristic curves (ICC) display the relationship between observed and expected responses at various levels of the measured variable (in this case dysautonomia). These differences are also quantified and expressed as standardized fit residuals with an expected value of 0 and an acceptable range between -2.5 and +2.5. Finally, the comparison of observed and expected responses can be formalized through an approximate chi-square statistic.

DIF is an additional aspect of model fit that concerns whether items work invariantly in different subgroups of respondents, e.g., age and gender groups [6, 13]. DIF may be uniform or non-uniform. When the magnitude of DIF is constant along the latent trait (e.g., levels of autonomic dysfunction), it is referred to as uniform DIF, whereas non-uniform DIF represents an interaction effect between group (e.g., gender) and location on the latent trait. No DIF means that the item works invariantly in both groups (e.g., men and women). DIF was tested by 2-way ANOVA of the residuals across autonomic dysfunction levels (subgroups of people with similar SCOPA-AUT scores) for time of assessment (baseline vs. follow-up), age (<69 vs. 69+) and gender. In case of uniform DIF, this can be adjusted for by splitting the affected item into subgroup specific items [6, 14]. Potential DIF-induced group-level bias was explored by estimating effect sizes of the differences between the person locations (logit measures) from non-adjusted and DIF-adjusted total scores. Items without DIF in the original scale were first anchored by their item locations from the DIF-adjusted scale to assure that the two sets of person estimates were on the same metric. Intraclass correlation coefficient and effect sizes (ES; mean difference divided by the overall standard deviation [15]) were calculated and used as indicators of the practical meaning and bias caused by any detected DIF. ESs of 0.2, 0.5, and 0.8 were regarded as small, moderate and large, respectively [16].

To assess ***local dependency***, relative correlations between standardized item residuals were examined [17, 18]. Local independence can be violated through response dependency (item redundancy) and trait dependency (multidimensionality) [19]. Response dependency occurs when the answer to one item governs the response to another because of similarities, e.g., if two or several items relate to the same aspect of the variable, whereas multidimensionality occurs when one or several items represent a different construct than the scale as a whole [19]. Local dependency can lead to inflated estimates of reliability and problems with construct validity. When assessing local dependency, individual residual correlations should be considered relative to the average observed residual correlation, rather than to a uniform value [18] . Residual correlations that are high, relative to the overall set of correlations, indicate violation of the local independence assumption [18]. The critical value for relative residual correlations was identified as described by Christensen et al. [17].

In circumstances where total scores represent a composition of subscales some local dependency will be expected between items addressing the same domain (i.e., items within the same subscales). For example, in the case of the SCOPA-AUT, its six subscales capture the complexity of dysautonomia and increases the validity of the scale beyond what would be achieved if only one aspect was represented [20, 21]. Therefore, we took account of the subscale structure of the SCOPA-AUT in the analysis by combining items within each domain into a subtest. In effect, this means that each subtest is treated as a single item in the analysis. For example, the 3-item cardiovascular SCOPA-AUT domain is treated as a single item with 10 response categories instead of as three unique 4-category items. This preserves the total domain score (9 in the case of the cardiovascular SCOPA-AUT domain) while absorbing the response dependency in the analysis [1]. Response dependence will be indicated if the reliability estimate from the analysis with six SCOPA-AUT subscales drops considerably compared to that from the SCOPA-AUT total scale based on all 23 separate items [18]. A subtest analysis absorbs local dependency, and the indices *A*, *c*, and *r* are estimated specific to the subtest

structure. The value *A* describes the non-error variance common to all subscales, *c* characterizes the variance that is unique to the subscales, and *r* is the latent correlation between the subscales. A subtest analysis performed on an approximate unidimensional scale will return a high value for both *A* and *r*, and a low value for *c* [20, 22].

References

1. Andrich D, Marais I. A course in Rasch measurement theory: measuring in the educational, social and health sciences: Springer; 2019.
2. Hobart J, Cano S: Improving the evaluation of therapeutic interventions in multiple sclerosis: the role of new psychometric methods. Health Technol Assess. 2009;13(12):iii, ix-x: 1-177.
3. Rasch G. Probabilistic Models for Some Intelligence and Attainment Tests. Copenhagen: Danish Institute for Educational Research; 1960.
4. Andrich D. Rasch models for measurement. Beverley Hills, CA: Sage Publications; 1988.
5. Ewing M, Salzberger T, Sinkovics R: An alternative approach to assessing cross-cultural measurement equivalence in advertising research. J Advert. 2005;34(1):17-36.
6. Hagquist C, Bruce M, Gustavsson JP. Using the Rasch model in nursing research: an introduction and illustrative example. Int J Nurs Stud 2009, 46(3):380-393.
7. Kyngdon A. Is Combining Samples Productive? A Quick Check via Tests of DIF. Rasch Meas Trans. 2011; 25(2):1324-1325.
8. Andrich D. An Index of Person Separation in Latent Trait Theory, the Traditional KR-20 Index, and the Guttman Scale Response Pattern. Educ Psychol Res 1982, 9(1):95-104.
9. Smith EV, Jr. Evidence for the reliability of measures and validity of measure interpretation: a Rasch measurement perspective. J Appl Meas. 2001;2(3):281-311.
10. Wright B, Masters G. Rating scale analysis. Chicag: MESA Press; 1982.
11. Schumacker R, Smith E: Reliability. A rasch perspective. Educ Psychol Meas. 2007;67(3):394-409.
12. Andrich D. Rating scales and Rasch measurement. Expert Rev Pharmacoecon Outcomes Res 2011, 11(5):571-585.
13. Hagquist C, Andrich D. Recent advances in analysis of differential item functioning in health research using the Rasch model. Health Qual Life Outcomes 2017, 15(1):181.
14. Brodersen J, Meads D, Kreiner S, Thorsen H, Doward L, McKenna S. Methodological aspects of Differential Item Functioning in the Rasch Model. J Med Econ. 2007;10:309-324.
15. Lipsey MW, Wilson DB. Practical meta-analysis. Thousand Oaks, Calif.: Sage; 2001.
16. Cohen J. Statistical power analysis for the behavioral sciences, 2. rev. edition. edn. Hillsdale N.J.: Lawrence Erlbaum Associates; 1988.
17. Christensen KB, Makransky G, Horton M. Critical Values for Yen’s Q3: Identification of Local Dependence in the Rasch Model Using Residual Correlations. Appl Psychol Meas. 2017;41(3):178-194.
18. Marais I. Local Dependence. In: Rasch Models in Health. Edited by Christensen K, Kreiner S, Mesbah M. John Wiley & Sons, Inc.; 2013: 111-130.
19. Marais I, Andrich D. Formalizing dimension and response violations of local independence in the unidimensional Rasch model. J Appl Meas. 2008;9(3):200-215.
20. Andrich D. Interpreting RUMM2030 Part IV: Multidimensionality and Subtests in RUMM. In. RUMM Laboratory, Perth, Western Australia; 2013.
21. Andrich D. Components of Variance of Scales With a Bifactor Subscale Structure From Two Calculations of alpha. Educ Meas-Issues Pra. 2016;35(4):25-30.
22. Guttersrud O, Naigaga MD, Pettersen KS. Measuring Maternal Health Literacy in Adolescents Attending Antenatal Care in Uganda: Exploring the Dimensionality of the Health Literacy Concept Studying a Composite Scale. J Nurs Meas. 2015;23(2):50E-66.
